# Running Phthalates to Ground: Pinpointing Exposure Sources in a Virtual Home

**DOI:** 10.1289/ehp.118-a80a

**Published:** 2010-02

**Authors:** Naomi Lubick

**Affiliations:** **Naomi Lubick** is a freelance science writer based in Zürich, Switzerland, and Folsom, California. She has written for *Environmental Science & Technology, Nature, and Earth*

Methods to measure concentrations of chemicals in adults and children, a science known as biomonitoring, can be costly and burdensome. And, although biomonitoring data provide useful aggregate information on exposure to all sources, it is almost impossible to tell how much comes from a specific source. A new mechanistic model may offer a way to identify the strongest sources of exposure to semivolatile organic compounds by showing how chemicals move from a single product through a home and which model parameters have the greatest influence on exposure **[*EHP*** **118:253–258; Xu et al.]**.

Researchers created a model of a hypothetical three-room house equipped with adjustable airflow systems to illustrate how human exposure to phthalates released by a specific source—in this case, vinyl flooring—might be predicted. Phthalates are plasticizers that are used in products as diverse as nail polish, plastic wiring, and children’s toys. Data from the Centers for Disease Control and Prevention suggest that more than three-quarters of the U.S. population may be exposed to these suspected endocrine disruptors.

The research team built on an earlier model that described how diethylhexyl phthalate (DEHP)—one of the most prevalent phthalates—is released from vinyl flooring into air and sorbs strongly to interior surfaces (walls, ceilings, floors, furniture, etc.) and suspended particles. Here the researchers used the model to explore the relative importance of inhalation of vapor, inhalation of particles, dermal sorption of DEHP, and oral ingestion of household dust on total exposure levels. To test which parameters might change the amount of total DEHP exposure through different routes, the researchers varied model parameters such as the amount of ventilation and velocity of air moving through their model house.

For example, they calculated that a fan pushing air through the house would cause more skin contact with phthalates by increasing the release rate from the vinyl surfaces to the air. The fan also thinned the layer of air cushioning the skin, increasing the transfer of DEHP from air to skin. Stagnant air without the fan caused less transfer of DEHP from air to skin, thus protecting against dermal uptake of DEHP.

The new model suggests that levels of phthalates measured in adults and children may result in part from contact with surfaces that may absorb high concentrations of DEHP, such as clothing. Changing the variables in the model house—from airflow to the amount of DEHP in the vinyl flooring to square footage in a room—made a difference in estimated exposure levels. Varying the parameters in this simple model demonstrated the potential for DEHP exposures arising from a single product to differ by as much as 40 times from one situation to another. That variability underscores the wide range of possible exposures across the population—and the difficulty of relying on biomonitoring alone to identify the most harmful sources.

## Figures and Tables

**Figure:**
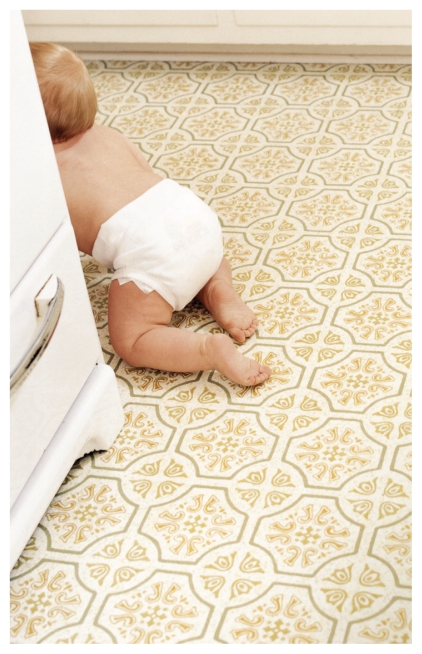
Exposure to gas-phase phthalates from vinyl flooring can occur through ingestion, inhalation, or dermal absorption.

